# Dosimetric evaluation and radioimmunotherapy of anti-tumour multivalent Fab́ fragments

**DOI:** 10.1038/sj.bjc.6690795

**Published:** 1999-11

**Authors:** J L Casey, R B Pedley, D J King, A J Green, G T Yarranton, R H J Begent

**Affiliations:** Cancer Research Campaign Targeting and Imaging Group, Department of Oncology, Royal Free and University College Medical School, Rowland Hill Street, London, NW3 2PF, UK; Celltech Therapeutics Ltd, Slough, Berkshire, UK

**Keywords:** dosimetry, radioimmunotherapy, tumour targeting, divalent Fab́, tri-valent Fab́

## Abstract

We have been investigating the use of cross-linked divalent (DFM) and trivalent (TFM) versions of the anti-carcinoembryonic antigen (CEA) monoclonal antibody A5B7 as possible alternatives to the parent forms (IgG and F(ab́)_2_) which have been used previously in clinical radioimmunotherapy (RIT) studies in colorectal carcinoma. Comparative biodistribution studies of similar sized DFM and F(ab́)_2_ and TFM and IgG, radiolabelled with both ^131^I and ^90^Y have been described previously using the human colorectal tumour LS174T nude mouse xenograft model (Casey et al (1996) *Br J Cancer* **74**: 1397–1405). In this study quantitative estimates of radiation distribution and RIT in the xenograft model provided more insight into selecting the most suitable combination for future RIT. Radiation doses were significantly higher in all tissues when antibodies were labelled with ^90^Y. Major contributing organs were the kidneys, liver and spleen. The extremely high absorbed dose to the kidneys on injection of ^90^Y-labelled DFM and F(ab́)_2_ as a result of accumulation of the radiometal would result in extremely high toxicity. These combinations are clearly unsuitable for RIT. Cumulative dose of ^90^Y-TFM to the kidney was 3 times lower than the divalent forms but still twice as high as for ^90^Y-IgG. TFM clears faster from the blood than IgG, producing higher tumour to blood ratios. Therefore when considering only the tumour to blood ratios of the total absorbed dose, the data suggests that TFM would be the most suitable candidate. However, when corrected for equitoxic blood levels, doses to normal tissues for TFM were approximately twice the level of IgG, producing a two-fold increase in the overall tumour to normal tissue ratio. In addition RIT revealed that for a similar level of toxicity and half the administered activity, ^90^Y-IgG produced a greater therapeutic response. This suggests that the most promising A5B7 antibody form with the radionuclide ^90^Y may be IgG. Dosimetry analysis revealed that the tumour to normal tissue ratios were greater for all ^131^I-labelled antibodies. This suggests that ^131^I may be a more suitable radionuclide for RIT, in terms of lower toxicity to normal tissues. The highest tumour to blood dose and tumour to normal tissue ratio at equitoxic blood levels was ^131^I-labelled DFM, suggesting that ^131^I-DFM may be best combination of antibody and radionuclide for A5B7. The dosimetry estimates were in agreement with RIT results in that twice the activity of ^131^I-DFM must be administered to produce a similar therapeutic effect as ^131^I-TFM. The toxicity in this therapy experiment was minimal and further experiments at higher doses are required to observe if there would be any advantage of a higher initial dose rate for ^131^I-DFM. © 1999 Cancer Research Campaign


*British Journal of Cancer* (1999) **81**(6), 972–980
© 1999 Cancer Research Campaign
Article no. bjoc.1999.0795

### Dosimetric evaluation and radioimmunotherapy of anti-tumour multivalent Fab´ fragments

**JL Casey[1], RB Pedley[1], DJ King[2]*, AJ Green[1], GT Yarranton[2]* and RHJ Begent[1]**

[1]Cancer Research Campaign Targeting and Imaging Group, Department of Oncology, Royal Free and University College Medical School, Rowland Hill Street, London NW3 2PF, UK; [2]Celltech Therapeutics Ltd, Slough, Berkshire, UK

**Summary** We have been investigating the use of cross-linked divalent (DFM) and trivalent (TFM) versions of the anti-carcinoembryonic antigen (CEA) monoclonal antibody A5B7 as possible alternatives to the parent forms (IgG and F(ab´)$_2$) which have been used previously in clinical radioimmunotherapy (RIT) studies in colorectal carcinoma. Comparative biodistribution studies of similar sized DFM and F(ab´)$_2$ and TFM and IgG, radiolabelled with both $^{131}$I and $^{90}$Y have been described previously using the human colorectal tumour LS174T nude mouse xenograft model (Casey et al (1996) *Br J Cancer* **74**: 1397–1405). In this study quantitative estimates of radiation distribution and RIT in the xenograft model provided more insight into selecting the most suitable combination for future RIT. Radiation doses were significantly higher in all tissues when antibodies were labelled with $^{90}$Y. Major contributing organs were the kidneys, liver and spleen. The extremely high absorbed dose to the kidneys on injection of $^{90}$Y-labelled DFM and F(ab´)$_2$ as a result of accumulation of the radiometal would result in extremely high toxicity. These combinations are clearly unsuitable for RIT. Cumulative dose of $^{90}$Y-TFM to the kidney was 3 times lower than the divalent forms but still twice as high as for $^{90}$Y-IgG. TFM clears faster from the blood than IgG, producing higher tumour to blood ratios. Therefore when considering only the tumour to blood ratios of the total absorbed dose, the data suggests that TFM would be the most suitable candidate. However, when corrected for equitoxic blood levels, doses to normal tissues for TFM were approximately twice the level of IgG, producing a two-fold increase in the overall tumour to normal tissue ratio. In addition RIT revealed that for a similar level of toxicity and half the administered activity, $^{90}$Y-IgG produced a greater therapeutic response. This suggests that the most promising A5B7 antibody form with the radionuclide $^{90}$Y may be IgG. Dosimetry analysis revealed that the tumour to normal tissue ratios were greater for all $^{131}$I-labelled antibodies. This suggests that $^{131}$I may be a more suitable radionuclide for RIT, in terms of lower toxicity to normal tissues. The highest tumour to blood dose and tumour to normal tissue ratio at equitoxic blood levels was $^{131}$I-labelled DFM, suggesting that $^{131}$I-DFM may be best combination of antibody and radionuclide for A5B7. The dosimetry estimates were in agreement with RIT results in that twice the activity of $^{131}$I-DFM must be administered to produce a similar therapeutic effect as $^{131}$I-TFM. The toxicity in this therapy experiment was minimal and further experiments at higher doses are required to observe if there would be any advantage of a higher initial dose rate for $^{131}$I-DFM. © 1999 Cancer Research Campaign

**Keywords**: dosimetry; radioimmunotherapy; tumour targeting; divalent Fab´; tri-valent Fab´

Tumour targeting using radiolabelled antibodies for radioimmunodetection (RID) and radioimmunotherapy (RIT) has been studied for many years. The clinical success of antibody-directed cancer treatment of common epithelial cancers has, however, been limited by low tumour uptake, immunogenicity and poor therapeutic ratios. In this paper we have used recombinant and chemical cross-linking technologies to develop a series of novel fragments (King et al, 1994, 1995; Antoniw et al, 1996; Casey et al, 1996), that have shown improved biodistribution properties and potential for future RID and RIT studies when compared to more conventional antibodies. These fragments consist of divalent and trivalent Fab´ fragments referred to as DFM and TFM, which can be synthesized from murine or humanized/chimaeric Fabs using mild conjugation techniques. The chemical cross-linkers have been designed to allow site-specific radiolabelling using $^{90}$Yttrium ($^{90}$Y), a high energy β-emitter.

It is important to match the most suitable isotope to each engineered antibody in order to achieve maximum therapeutic benefit. $^{131}$I has been the radionuclide used in the majority of RIT studies to date because of its ready availability, low cost, simple conjugation and effectiveness in responses in clinical RIT studies. However, there are several limitations that have prompted the search for a more suitable radiolabel. The high level of γ-emission has led to problems with patient handling, waste disposal and contributes to myelosuppression. $^{90}$Y is a pure β-emitter with a comparatively short half-life and thus $^{90}$Y-labelled antibodies may be an attractive alternative.

Using the murine monoclonal anti-CEA antibody A5B7 and its F(ab´)$_2$ fragment radiolabelled with $^{131}$I, we have regularly produced impressive growth regression and complete irradication of tumours in nude mice bearing human colorectal tumour xenografts (Pedley et al, 1993, 1996). However, clinical trials using high doses of $^{131}$I-A5B7 (Lane et al, 1994), have produced a response rate of only 10%, although this is in line with other colorectal antibodies used for RIT and some of the best chemotherapeutic agents such as 5-fluorouracil (5-FU). As a result

*Received 1 March 1999*
*Revised 19 May 1999*
*Accepted 21 May 1999*
*Correspondence to:* RB Pedley

*Present address: Coulter Pharmaceuticals, 550 California Avenue, Palo Alto, CA 94306–1440, USA

**Table 1** Biodistribution of $^{131}$I- and $^{90}$Y-labelled A5B7 F(ab´)$_2$, DFM, IgG and TFM in mice bearing LS174T human tumour xenografts

| | $^{131}$I-F(ab´)$_2$ | | | | $^{131}$I-DFM | | | | $^{90}$Y-F(ab´)$_2$ | | | | $^{90}$Y-DFM | | | |
|---|---|---|---|---|---|---|---|---|---|---|---|---|---|---|---|---|
| | 3 h | 24 h | 48 h | 144 h | 3 h | 24 h | 48 h | 144 h | 3 h | 24 h | 48 h | 144 h | 3 h | 24 h | 48 h | 144 h |
| Blood | 13.3 | 0.25 | 0.09 | 0.01 | 10.4 | 0.30 | 0.12 | 0.02 | 10.1 | 0.41 | 0.30 | 0.19 | 7.24 | 0.19 | 0.29 | 0.15 |
| Liver | 4.63 | 0.15 | 0.05 | 0.01 | 3.26 | 0.15 | 0.06 | 0.01 | 5.10 | 7.75 | 3.12 | 0.64 | 11.1 | 8.25 | 6.30 | 0.92 |
| Kidney | 7.96 | 0.23 | 0.12 | 0.02 | 6.97 | 0.20 | 0.12 | 0.03 | 23.9 | 27.0 | 17.8 | 2.86 | 31.0 | 27.6 | 15.9 | 1.97 |
| Lung | 7.07 | 0.37 | 0.10 | 0.02 | 5.99 | 0.36 | 0.17 | 0.03 | 5.11 | 1.14 | 1.27 | 0.57 | 4.06 | 0.84 | 2.35 | 0.54 |
| Spleen | 4.56 | 0.25 | 0.10 | 0.03 | 4.16 | 0.30 | 0.10 | 0.09 | 4.84 | 8.37 | 4.77 | 1.94 | 9.73 | 11.2 | 11.4 | 3.77 |
| Colon | 2.20 | 0.10 | 0.04 | 0.02 | 1.67 | 0.13 | 0.07 | 0.04 | 1.54 | 1.46 | 1.36 | 0.32 | 1.83 | 1.70 | 1.45 | 0.34 |
| Muscle | 1.43 | 0.12 | 0.12 | 0.01 | 0.98 | 0.13 | 0.22 | 0.04 | 0.72 | 0.62 | 0.48 | 0.29 | 0.55 | 0.56 | 0.55 | 0.21 |
| Femur | – | – | – | – | – | – | – | – | 1.86 | 1.59 | 2.17 | 0.61 | 3.44 | 2.74 | 2.23 | 0.87 |
| Tumour | 9.67 | 9.15 | 3.72 | 1.50 | 10.6 | 7.40 | 4.41 | 1.03 | 9.32 | 9.19 | 6.38 | 0.91 | 6.66 | 6.81 | 6.62 | 1.25 |

| | $^{131}$I-IgG | | | | $^{131}$I-TFM | | | | $^{90}$Y-IgG | | | | $^{90}$Y-TFM | | | |
|---|---|---|---|---|---|---|---|---|---|---|---|---|---|---|---|---|
| | 3 h | 24 h | 48 h | 144 h | 3 h | 24 h | 48 h | 144 h | 3 h | 24 h | 48 h | 144 h | 3 h | 24 h | 48 h | 144 h |
| Blood | 33.5 | 9.81 | 3.32 | 0.46 | 24.2 | 2.71 | 0.64 | 0.06 | 19.7 | 5.28 | 3.09 | 0.36 | 19.1 | 1.08 | 0.33 | 0.04 |
| Liver | 9.25 | 3.88 | 0.74 | 0.23 | 6.50 | 0.93 | 0.38 | 0.07 | 6.10 | 7.63 | 8.71 | 2.83 | 12.1 | 7.02 | 5.29 | 0.97 |
| Kidney | 6.58 | 2.19 | 1.13 | 0.29 | 6.80 | 1.01 | 0.48 | 0.10 | 6.53 | 3.65 | 1.61 | 0.71 | 8.62 | 6.12 | 5.11 | 1.28 |
| Lung | 15.2 | 4.76 | 2.10 | 0.41 | 8.23 | 2.10 | 0.70 | 0.11 | 8.81 | 3.12 | 2.14 | 0.86 | 10.1 | 2.04 | 1.78 | 0.47 |
| Spleen | 9.10 | 3.03 | 0.79 | 0.36 | 7.12 | 1.13 | 0.61 | 0.18 | 5.65 | 4.32 | 2.90 | 1.99 | 8.20 | 8.19 | 5.83 | 1.28 |
| Colon | 2.08 | 1.11 | 0.47 | 0.23 | 2.02 | 0.63 | 0.39 | 0.13 | 2.17 | 1.59 | 0.88 | 0.58 | 2.18 | 2.04 | 1.19 | 0.42 |
| Muscle | 1.21 | 1.23 | 0.45 | 0.21 | 0.95 | 0.74 | 0.35 | 0.18 | 0.97 | 0.89 | 0.51 | 0.37 | 0.81 | 1.26 | 0.74 | 0.42 |
| Femur | – | – | – | – | – | – | – | – | 1.68 | 1.90 | 1.28 | 0.76 | 2.52 | 3.24 | 2.41 | 0.87 |
| Tumour | 8.73 | 19.6 | 14.5 | 6.25 | 11.2 | 15.1 | 8.80 | 2.38 | 8.28 | 23.0 | 16.6 | 4.43 | 7.32 | 16.8 | 10.8 | 1.75 |

Time points at 3 h, 24 h, 48 h and 144 h after intravenous injection. Results are expressed as percentage of injected activity per gram of tissue (median of four mice per time point) and decay corrected using the decay constants for $^{131}$I and $^{90}$Y.

we have been looking at various ways to improve the use of this antibody in future RIT clinical trials.

A recent study was designed to compare different forms of the murine antibody A5B7 raised against carcinoembryonic antigen (CEA), in order to determine which was the optimal form of the antibody for RIT with the isotopes $^{131}$I and $^{90}$Y (Casey et al, 1996). Different forms of murine A5B7, namely DFM, F(ab´)$_2$, IgG and TFM were synthesized and the functional affinities for CEA were measured under similar conditions using surface plasmon resonance. The study revealed that there was a significantly faster association rate for DFM compared to the other antibody forms. TFM had the slowest dissociation rate; however, this was not significantly different to the divalent antibody forms. This is probably a limitation of the analysis in measuring very slow dissociation rates, since other similar studies have shown clearly that TFM forms of other antibodies show a significant advantage in terms of a slower dissociation rate (King et al, 1994, 1995). These improved kinetic binding characteristics of cross-linked DFM and TFM may have implications for improved RIT. Biodistribution experiments were also performed in this study to compare similar sized F(ab´)$_2$ and DFM, and IgG and TFM with both $^{131}$I and $^{90}$Y (Casey et al, 1996). Results indicated the cross-linked antibodies were highly stable in vivo and that $^{131}$I-DFM and $^{90}$Y-TFM would be the most suitable combinations of antibody and radionuclide for RIT with A5B7.

When selecting new possible candidates for RIT, it is important to consider not only the biodistribution data at various time points after injection, but also quantitative measurement of cumulative radiation dose to tumour and normal tissues with respect to time. Although these dosimetry calculations are based on data generated from a murine model, and can be considered only as estimates, they have previously proved to be useful predictors of toxicity and a guide to the levels of activity that may be administered for RIT (Pedley et al, 1989, 1993).

The results for this previous study were based only on functional affinity and biodistribution data of both $^{131}$I and $^{90}$Y-labelled F(ab´)$_2$, DFM, IgG and TFM. In this second study, detailed dosimetry analysis of the biodistribution data is described in similar comparative experiments. These calculations were useful in predicting the relative radiation doses that could be administered for RIT, and an indication of the resulting toxicity.

### MATERIALS AND METHODS

### Preparation and characterization of DFM and TFM

Details of preparation and characterization of murine A5B7 DFM and TFM have been described previously in detail by Casey et al (1996). Briefly F(ab´)$_2$ was partially reduced with 2-mercaptoethylamine to form Fab´ fragments and produce a free hinge thiol for cross-linking. After desalting to remove free reducing agent, a 2:1 molar excess of Fab´ to dimaleimide CT52 cross-linker and a 3:1 molar excess of Fab´ to trimaleimide CT998 cross-linker was added and the reaction left to proceed at 37°C for 2 h. Final purification of DFM and TFM was performed by high-performance liquid chromatography (HPLC) gel filtration.

### Dosimetry

The estimated total doses of β-radiation (per MBq injected) to the blood, tumour and normal tissues were evaluated for each antibody-radiolabel conjugate using the following calculations. Figures for percentage of injected activity per gram of each tissue (% ia g$^{-1}$) for individual mice were derived by direct measurement

*© 1999 Cancer Research Campaign* 

of blood and tissues using a γ counter, and the average median value was calculated for each time point as described in detail in a previous publication (Casey et al, 1996). These values were decay corrected using the following equation and figures shown in Table 1.

$A = A_0 e^{-\lambda t}$ where $\lambda = \mathrm{Ln}2/t_{1/2}$
t = time after injection
$A_0$ = original activity at time t
A = activity at time t after decay correction
$t_{1/2}$ = half-life of 194 h for $^{131}$I and 64 h for $^{90}$Y.

The area under the % ia $g^{-1}$ over time curve (AUC) was calculated using the trapezoidal rule assuming no activity in the tumour at time 0, and 40% in the blood at time 0. This value for blood was generated from an experiment with both $^{131}$I-labelled A5B7 IgG and F(ab´)$_2$, in which blood samples were counted at 30 s post-injection. An average of 39% IgG and 40.7% F(ab´)$_2$ % ia $g^{-1}$ was present in the blood at this early time point. This is consistent with the estimated total blood volume of a 20–30 g mouse being in the range 2.0–2.5 ml as described by Durbin et al (1992). The total decay corrected AUC was converted from % ia $g^{-1}$ per μCi to MBq $g^{-1}$ using the following calculation: AUC per 100% × 1000 (for mCi) per 37 (for MBq).

To calculate total β dose to individual organs the MIRD S factor of 0.369 for $^{131}$I and 1.93 for $^{90}$Y (MIRD Pamphlet 11, 1975) was used to convert MBq $g^{-1}$ to cGy $h^{-1}$. This calculation does not take into account the contribution of γ energy as most of this penetrating radiation escapes the mouse and there is virtually no self-absorption of γ rays. The calculation also assumes that there is uniform distribution of activity, and there were no cross-organ β radiation doses.

Total β doses to tumour and individual organs were normalized to an equal dose of 100 cGy to the blood. This was achieved by correcting the blood dose to 100 cGy then dividing or multiplying the tissue doses by this correction factor. For example the blood dose of 23 cGy for $^{131}$I-F(ab´)$_2$ is multiplied by 4.35 to equal 100 cGy, the radiation doses to tumour and normal tissues are multiplied by 4.35 to give the estimated absorbed dose at 100 cGy.

### Radioimmunotherapy

To assess the therapeutic potential of DFM and TFM, RIT experiments in the nude mouse xenograft model were designed. The estimated dosimetry calculations were used to predict the total activity of radiolabelled antibodies that should be administered for RIT.

DFM and TFM were radiolabelled with $^{131}$I using the chloramine T method. Free iodine was removed using a PD-10 coloumn, and the percentage incorporation of radiolabel was analysed by thin-layer chromatography (TLC) analysis in 80% methanol. Antigen binding analysis was performed to ensure immunoreactivity was retained, by applying a dilution of the radiolabelled antibody to a 1-ml CEA affinity column and measuring the percentage bound, as described previously by Casey et al (1995).

Therapy experiments were performed using the same nude mouse model used for the biodistribution experiment described previously (Casey et al, 1996). Experiments commenced when the LS174T human colon carcinoma xenograft tumours were between 0.1–0.2 cm$^3$ in volume and in exponential growth (7–10 days after passaging). 18.5 MBq $^{131}$I-TFM and 37 MBq $^{131}$I-DFM were injected intravenously via the tail vein into six mice per group, and a further six untreated mice were used as a control group. Tumours were measured and blood samples were taken (four mice per group) from anaesthetized mice at regular intervals until the white blood cell count (WBC) returned to normal and the tumour volume reached 2 cm$^3$, when mice were sacrificed. Four additional mice per group were used for biodistribution, one-tenth of the dose for RIT was administered and 24 h later blood, tumour and tissue samples (liver, kidney, lung, spleen, colon, muscle, femur) were removed for activity assessment in the γ counter.

TFM and IgG were radiolabelled with $^{90}$Y using the method described previously (Casey et al, 1996). Incorporation was measured by TLC in 0.1 M citrate buffer pH 5.0, and HPLC gel filtration was used to remove any free $^{90}$Y. For RIT 11.1 MBq $^{90}$Y-TFM and 5.6 MBq $^{90}$Y-IgG was injected into six mice per group, six mice were left untreated as a control group. A further four mice were used for a biodistribution experiment as for the $^{131}$I therapy. Tumour measurement and white cell blood counting was also performed as described above.

Statistical comparisons were performed using the Mann–Whitney *U*-test for non-parametric statistical analysis.

**Table 2** Estimated tissue doses of β-radiation (cGy) per MBq injected activity calculated from the total area under the curve (AUC) using the trapezoidal rule, from the biodistribution data presented in Table 1

| Tissue | $^{131}$I-F(ab´)$_2$ | $^{131}$I-DFM | $^{90}$Y-F(ab´)$_2$ | $^{90}$Y-DFM |
|---|---|---|---|---|
| Blood | 23.0 | 20.0 | 114 | 91.6 |
| Liver | 6.22 | 4.59 | 236 | 387 |
| Kidney | 10.8 | 9.73 | 1084 | 1065 |
| Lung | 10.0 | 9.19 | 99.2 | 122 |
| Spleen | 6.76 | 6.76 | 327 | 643 |
| Colon | 3.24 | 2.97 | 77.3 | 85.4 |
| Muscle | 2.70 | 2.97 | 34.1 | 32.7 |
| Femur | – | – | 113 | 145 |
| Tumour | 61.4 | 60.5 | 389 | 360 |
| Tumour/blood ratio | 2.67 | 3.03 | 3.41 | 3.93 |

**Table 3** Estimated tissue doses of β-radiation (cGy) per MBq injected activity calculated from the total area under the curve (AUC) using the trapezoidal rule, from the biodistribution data described in Table 1

| Tissue | $^{131}$I-IgG | $^{131}$I-TFM | $^{90}$Y-IgG | $^{90}$Y-TFM |
|---|---|---|---|---|
| Blood | 90.3 | 44.9 | 322 | 175 |
| Liver | 25.1 | 12.4 | 470 | 349 |
| Kidney | 20.8 | 13.8 | 152 | 316 |
| Lung | 43.2 | 19.2 | 180 | 155 |
| Spleen | 24.1 | 15.4 | 227 | 360 |
| Colon | 8.65 | 6.77 | 74.3 | 85.4 |
| Muscle | 7.84 | 5.68 | 41.6 | 53.5 |
| Femur | – | – | 91.9 | 151 |
| Tumour | 172 | 111 | 951 | 627 |
| Tumour/blood ratio | 1.90 | 2.48 | 2.95 | 3.58 |

### RESULTS

The data illustrated in Table 1 was generated from comparative biodistribution experiments of $^{131}$I- and $^{90}$Y-labelled A5B7 F(ab´)$_2$ and DFM, and $^{131}$I- and $^{90}$Y-labelled A5B7 IgG and TFM, which was described in detail previously (Casey et al, 1996). These figures have been corrected for radioactive decay and were used to calculate estimates of the total absorbed dose to blood, tumour and

 © 1999 Cancer Research Campaign

**Table 4** Radiation dose estimates to organs in nude mice given $^{131}$I- or $^{90}$Y-labelled A5B7 F(ab´)$_2$, DFM, IgG or TFM (Tables 2 and 3) normalized to a 100 cGy blood dose

| Tissue | $^{131}$I F(ab´)$_2$ | $^{131}$I DFM | $^{131}$I IgG | $^{131}$I TFM | $^{90}$Y F(ab´)$_2$ | $^{90}$Y DFM | $^{90}$Y IgG | $^{90}$Y TFM |
|---|---|---|---|---|---|---|---|---|
| Blood | 100 | 100 | 100 | 100 | 100 | 100 | 100 | 100 |
| Liver | 27.0 | 23.0 | 27.8 | 27.6 | 207 | 422 | 146 | 199 |
| Kidney | 47.0 | 48.7 | 23.0 | 30.7 | 951 | 1163 | 47.2 | 181 |
| Lung | 43.5 | 46.0 | 47.8 | 42.8 | 87.0 | 133 | 55.9 | 88.6 |
| Spleen | 29.4 | 33.8 | 26.7 | 34.3 | 287 | 702 | 70.5 | 206 |
| Colon | 14.1 | 14.9 | 9.60 | 15.1 | 67.8 | 93.2 | 23.1 | 48.8 |
| Muscle | 11.7 | 14.9 | 8.68 | 12.7 | 29.9 | 35.7 | 12.9 | 30.6 |
| Tumour | 267 | 303 | 191 | 247 | 341 | 393 | 295 | 358 |
| TEDNT | 173 | 181 | 144 | 163 | 1630 | 2549 | 356 | 754 |
| Ratio | 1.54 | 1.67 | 1.33 | 1.52 | 0.21 | 0.15 | 0.83 | 0.47 |

TEDNT refers to total estimated dose to normal tissues which is the sum of the dose estimates for liver, kidney, lung, spleen, colon and muscle. NB. Femur has not been included. Ratio refers to tumour to normal tissue (TEDNT) ratio.

normal tissues in the nude mouse LS174T human colorectal tumour xenograft model.

### Dosimetry

Total absorbed radiation doses were estimated by calculating the AUC for each antibody-radiolabel conjugate and using the MIRD correction factor to convert MBq to absorbed dose in cGy h$^{-1}$. These values are illustrated in Tables 2 and 3.

### *F(ab´)$_2$ and DFM*

The radiation doses and tumour to blood ratios were similar for F(ab´)$_2$ and DFM when each was labelled with the same isotope (Table 2). However, there was a wide discrepancy between the two radionuclides $^{131}$I and $^{90}$Y; tissue doses per MBq administered from $^{90}$Y-labelled antibody fragments were at least tenfold higher than those delivered by $^{131}$I-labelled antibody fragments.

A notable difference between the radionuclides is the high dose to the kidney on radiolabelling with $^{90}$Y (1065–1084 cGy), which is 100 times greater than for $^{131}$I-labelled F(ab´)$_2$ and DFM (9.73–10.8 cGy). In spite of its size, it is evident that a large proportion of antibody clears through and is retained by the kidney and the toxicity to this organ would severely limit the dose of $^{90}$Y that could be administered for therapy.

The dosimetry calculations also revealed higher absorbed doses for spleen (DFM: 643 cGy, F(ab´)$_2$: 327 cGy) and liver (DFM: 387 cGy, F(ab´)$_2$: 236 cGy) on administration of $^{90}$Y-DFM compared to $^{90}$Y-F(ab´)$_2$. There may be higher non-specific uptake of $^{90}$Y-DFM by the RES that could account for the increase in overall dose to these organs.

### *IgG and TFM*

As found for the F(ab´)$_2$ and DFM, the radiation dose delivered to all tissues per MBq $^{90}$Y-labelled antibodies was far greater than from the $^{131}$I-labelled antibodies. Radiation doses to blood, tumour and all normal organs were lower on administration of $^{131}$I-TFM compared to $^{131}$I-IgG (Table 3). This reflects the faster clearance of TFM from the circulation and normal tissues due to the lack of the Fc portion, which is responsible for the long half-life of IgG through FcRn-mediated recycling (Ghetie et al, 1997). However, this was not observed with $^{90}$Y-TFM; although blood and tumour levels were lower, doses to normal tissues were similar and sometimes greater than for $^{90}$Y-IgG. This is most likely due to retention of $^{90}$Y in cells for longer than $^{131}$I, as has been demonstrated previously (Press et al, 1996), which contributes largely to the toxicity.

The faster blood clearance of TFM compared to IgG is reflected here in the overall blood doses and tumour to blood ratios (Table 3). The bone marrow has been identified as the dose-limiting organ in RIT. The clearance rate of radioactivity from the blood has been suggested as a means of predicting bone marrow toxicity (Siegal et al, 1990), because dose to the marrow equals 0.2–0.4 times that of the blood. Therefore since the blood received approximately half the cumulative dose on administration of $^{131}$I-TFM or $^{90}$Y-TFM compared to IgG conjugates, this will result in a favourable twofold decrease in toxicity to the bone marrow.

Normal tissue toxicity for both iodine labelled IgG and TFM was low and the largest normal tissue dose excluding blood (bone marrow) was to the lungs. Histological studies have demonstrated that A5B7 IgG shows some cross-reactivity with lung tissue (Boxer et al., 1995), which is possibly due to the presence of low normal levels of CEA produced in the lungs.

The largest normal tissue doses for $^{90}$Y-IgG were to the liver and spleen and for $^{90}$Y-TFM the liver, kidney and spleen. The absorbed dose to the kidney was doubled for $^{90}$Y-TFM compared to $^{90}$Y-IgG, but was fourfold lower than for the smaller $^{90}$Y-labelled fragments. It is likely that a proportion of $^{90}$Y-TFM or fragments of $^{90}$Y-TFM are filtered by the kidney and cleared via this route, which may explain this increase in dose. Conversely, the liver dose for $^{90}$Y-TFM was lower than for $^{90}$Y-IgG which clears largely via the liver. A higher spleen dose was generated on administration of $^{90}$Y-TFM compared to $^{90}$Y-IgG, and this may be due to increased recognition by the RES. Absorbed dose to the bone was also higher for $^{90}$Y-TFM but this level was similar to the absorbed dose by other normal organs such as lung.

### *Comparative dosimetry of antibodies after blood normalization*

Radiation dose estimates provided in Table 2 and 3 were normalized to an equal dose (100 cGy) to the blood in Table 4. Since the levels of radioactivity in the blood has been suggested as a means of predicting bone marrow toxicity (Siegel et al, 1990) this Table, based on an equal blood dose, should reflect the estimated tumour dose and normal tissue toxicity at approximately equal myelotoxic levels.

*© 1999 Cancer Research Campaign* 

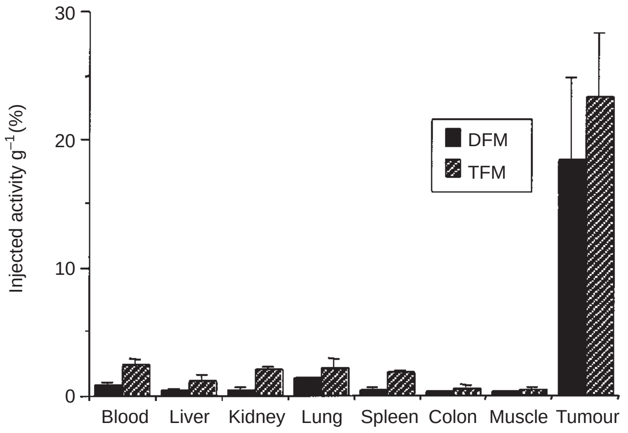

**Figure 1** Biodistribution of one-tenth of the administered activity for therapy of $^{131}$I-DFM and $^{131}$I-TFM 24 h after injection to nude mice bearing LS174T human colorectal tumour xenografts. Results are expressed as % of injected activity per gram of tissue, columns are a mean of four mice and bars represent standard deviations

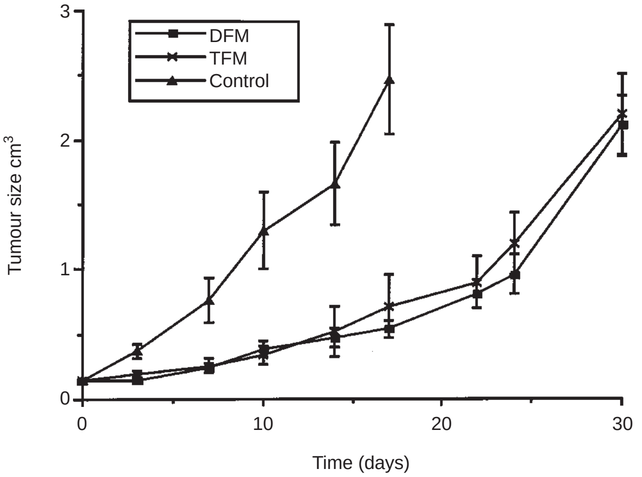

**Figure 2** Effect of treatment regimes on the growth of LS174T tumours with (A) $^{131}$I-DFM, (B) $^{131}$I-TFM and (C) no treatment control group. Vertical bars indicate standard error of the mean (s.e.m.); points are a mean of six mice

### *$^{90}$Y-labelled antibodies*

Referring to the total estimated toxicity doses to normal tissues (TEDNT) in Table 4, clearly the extremely high levels for $^{90}$Y-F(ab´)$_2$ and $^{90}$Y-DFM should exclude these combinations from further consideration for RIT. This limits the choice to either IgG or TFM labelled with $^{90}$Y, both of which are worthy of further consideration. For equitoxic levels to the blood a slightly higher tumour dose was estimated for $^{90}$Y-TFM, but the dose to normal tissues was 2.1 times higher than that of $^{90}$Y-IgG. TEDNT for $^{90}$Y-TFM was 2.1 times greater than the estimated dose to the tumour, whereas TEDNT was only 1.2 times greater than the tumour dose for $^{90}$Y-IgG. This suggests that for a given blood dose $^{90}$Y-IgG would be less toxic than $^{90}$Y-TFM. Thus a higher therapeutic tumour:normal tissue ratio was achieved for IgG, suggesting that RIT with the isotope $^{90}$Y would be least toxic by conjugation to IgG, allowing increased doses of isotope which may lead to more effective therapy.

### $^{131}$I-labelled antibodies

All combinations of antibodies labelled with $^{131}$I corrected for equitoxic blood levels produced similar TEDNT (range 144–281 cGy) and tumour doses (range 191–303 cGy). The highest tumour:normal tissue ratios were achieved for the smaller sized fragments DFM and F(ab´)$_2$ suggesting favourable therapeutic advantage over TFM and IgG. The highest tumour dose and tumour:normal tissue ratio overall was for DFM, implying that RIT with the isotope $^{131}$I would be most effective by conjugation to DFM.

### Radioimmunotherapy

### *RIT of $^{131}$I labelled DFM and TFM*

The dosimetry calculations revealed that for the same administered activity (MBq), the blood and tumour dose of $^{131}$I-TFM was approximately double that of $^{131}$I-DFM. It was therefore estimated that similar therapy and toxicity would be achieved by administering twice the amount of activity of $^{131}$I-DFM compared to $^{131}$I-TFM. The amount of activity to be administered for RIT studies was calculated with reference to therapeutic levels administered in previous RIT studies with A5B7 $^{131}$I-IgG and $^{131}$I-F(ab´)$_2$. RIT experiments demonstrated identical therapeutic response by administration of 18.5 MBq $^{131}$I-IgG and 37 MBq F(ab´)$_2$ (Pedley et al, 1993).

DFM was labelled with $^{131}$I to a specific activity of 0.37 MBq $\mu g^{-1}$ and TFM to 0.26 MBq $\mu g^{-1}$. TLC after desalting revealed 90% incorporation of $^{131}$I for TFM and 95% incorporation for DFM. Antigen binding analysis post-labelling resulted in retention of 75% binding of $^{131}$I-TFM and 86% of $^{131}$I-DFM. For RIT 18.5 MBq $^{131}$I-TFM and 37 MBq $^{131}$I-DFM was injected into six mice per group. Biodistribution at 24 h in Figure 1 demonstrated efficient tumour localization of both DFM and TFM.

Figure 2 shows the mean tumour growth following administration of therapeutic levels of $^{131}$I-DFM and $^{131}$I-TFM compared to a no treatment control group. Both DFM and TFM produced a significant therapeutic effect when compared with the control group ($P < 0.05$). Tumour growth was delayed for approximately 25 days for both conjugates, and there was no significant difference between the two therapy groups ($P > 0.05$).

Toxicity was assessed by measurement of white blood cell count (WBC) at weekly intervals until the WBC returned to normal levels (Figure 3). Little toxicity was produced by either DFM or TFM ($P > 0.05$) and for 2/4 mice from each group the WBC was unaffected after treatment. The remaining 2/4 mice treated with $^{131}$I-DFM and $^{131}$I-TFM showed similar toxicity, with a maximum fall in WBC of > 30% up to 14 days post-therapy. After 21 days the WBC returned to normal for both TFM and DFM (i.e. equivalent or greater than the original pre-therapy figure). Control mice also experienced a decrease in WBC when tumours were approaching 2 cm$^3$.

### *RIT of $^{90}$Y labelled IgG and TFM*

The blood radiation dose was doubled on administration of the same activity (MBq) of $^{90}$Y-IgG compared to $^{90}$Y-TFM. Assuming the same principle that toxicity is related to total blood dose which in turn reflects absorbed marrow dose, it was predicted that twice the amount of activity of TFM compared to IgG could be administered for the same level of toxicity. An RIT study was designed with reference to dosimetry evaluations and previous therapeutic levels of activity administered for $^{90}$Y-IgG of another antibody A33 (Antoniw et al, 1996).

© 1999 Cancer Research Campaign

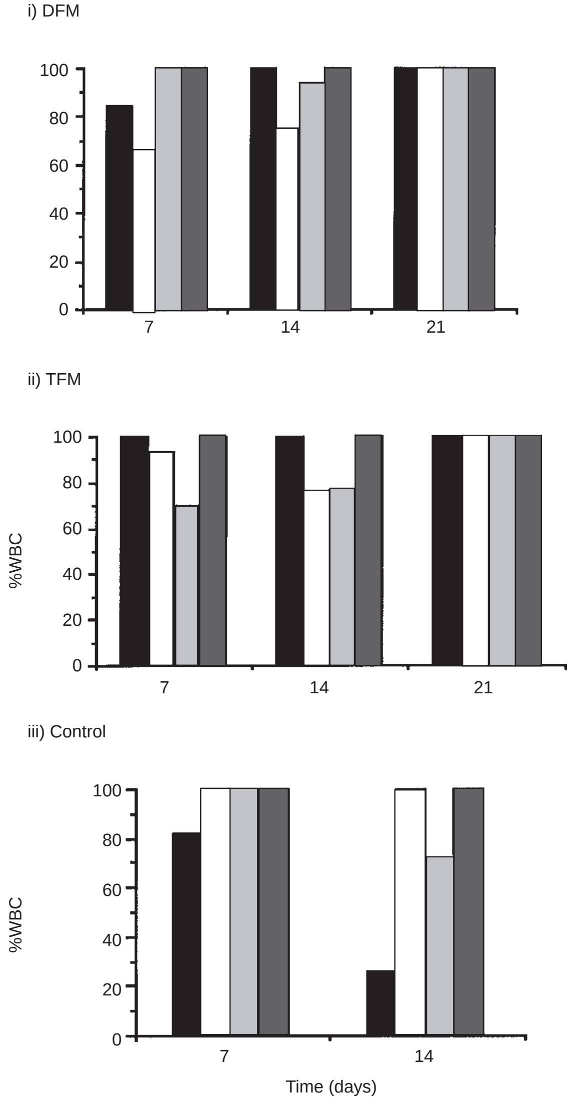

**Figure 3** White blood cell counts (WBC) measured weekly from four mice per group. Results are expressed as a % of the original pre-therapy WBC, for individual mice

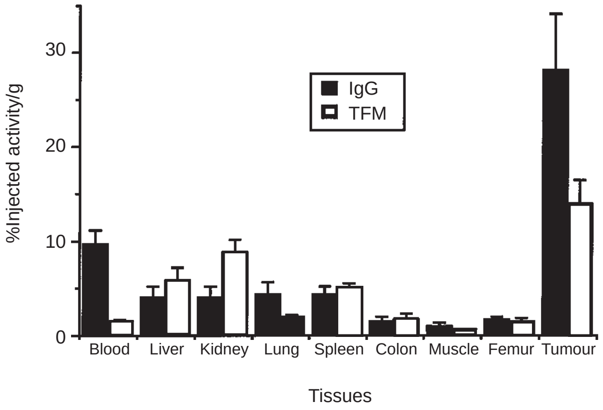

**Figure 4** Biodistribution of one-tenth of the dose administered for therapy of $^{90}$Y-IgG and $^{90}$Y-TFM 24 h after injection to nude mice bearing LS174T human tumour xenografts. Results are expressed as % of injected activity per gram of tissue, columns are a mean of four mice and bars represent standard deviations

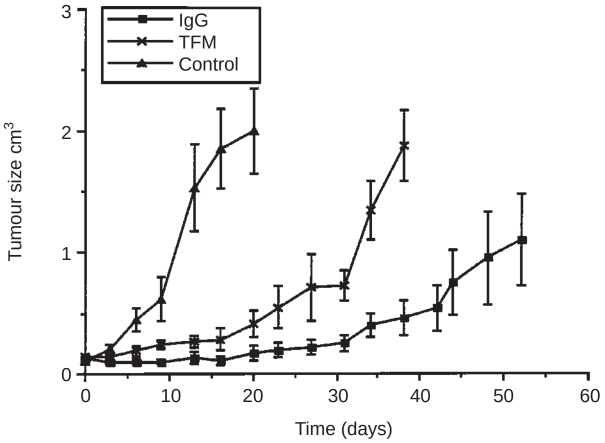

**Figure 5** Effect of treatment regimes on the growth of LS174T tumours with (A) $^{90}$Y-IgG, (B) $^{90}$Y-TFM and (C) no treatment control group. Vertical bars indicate s.e.m., points are a mean of six mice

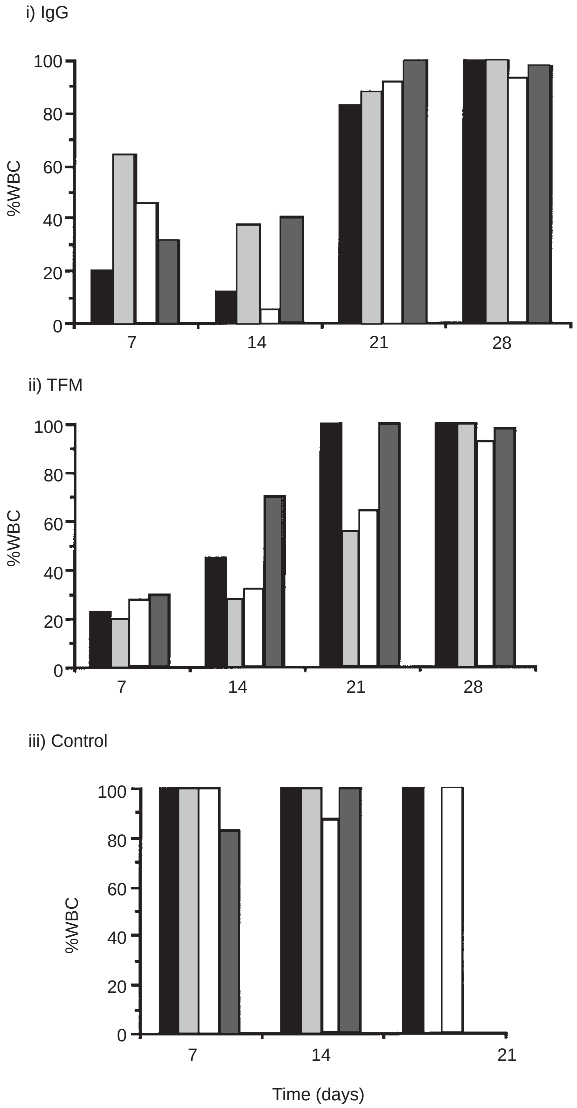

**Figure 6** White blood cell counts (WBC) measured weekly from four mice per group. Results are expressed as a % of the original pre-therapy WBC

IgG was labelled to a specific activity of 0.09 MBq μg$^{-1}$ and TFM to 0.15 MBq μg$^{-1}$. Free $^{90}$Y was removed by HPLC purification as described previously (Casey et al, 1996), and TLC analysis showed > 90% incorporation of radiolabel. Antigen binding analysis post-labelling resulted in 71% binding of IgG and 86% for TFM. Biodistribution at 24 h in Figure 4 demonstrated efficient tumour localization of both IgG and TFM.

Figure 5 illustrates the relative growth delay of $^{90}$Y-IgG and $^{90}$Y-TFM compared to a no treatment control group. Both IgG and TFM produced a significant therapeutic effect when compared with the control group ($P < 0.05$). Tumour growth was delayed for approximately 17 days for TFM and 31 days for IgG. The therapeutic response of $^{90}$Y-TFM was not as effective as $^{90}$Y-IgG despite administration of double the amount of activity.

Toxicity, assessed by WBC (Figure 6), was observed for all mice except the control group and lasted up to 21 days. Although predicted to be equal by dosimetry estimates, the overall toxicity was greatest for IgG, resulting in a fall of WBC in all four mice at 14 days to 40% (range 5–40%) of the pre-therapy WBC. For TFM the greatest fall in WBC was observed 7 days post-injection to 30% (range 20–30%). By 14 days recovery of WBC resulted in a return to 70% (range 28–70%) of the original value. At 21 days post-injection there was almost complete recovery of WBC, with the exception of 2/4 mice treated with $^{90}$Y-TFM, which had WBC of 56% and 64% of the original values. This slower return to normal WBC levels is most likely due to the higher dose administered, but there was no significant difference in WBC toxicity between IgG and TFM levels at each time point ($P > 0.05$).

### DISCUSSION

Chemically cross-linked divalent and trivalent versions of the murine antibody A5B7 have shown high stability both in vitro and in nude mice bearing human tumour xenografts (Casey et al, 1996). In this previous study the cross-linked antibodies showed potential as possible new candidates for RIT, with favourable affinity, biodistribution and tumour uptake coupled with the ability to humanize for reduced immunogenicity. The aim of this study was to measure quantitatively the dose to tumour and normal tissue and to use these estimates to predict the relative doses for use in therapeutic studies. To date only a few studies have considered measurement of radioactivity dose to tissues in model systems, and it is becoming clear that this preclinical data is very important in assessing the level of toxicity to normal tissues compared to the dose to tumour. This is particularly pertinent when the aim is to select optimal combinations of radionuclide and antibody for RIT.

The most prominent difference between the two radionuclides was the increase in dose to all tissues on labelling with $^{90}$Y (Tables 2 and 3). Blood doses were 4–6 times higher for divalent antibodies labelled with $^{90}$Y as opposed to $^{131}$I, and 3–4 times higher for TFM and IgG. Similarly tumour levels were five- to sixfold higher than for an equivalent injected dose of $^{131}$I-labelled antibodies. Several other publications (Sharkey et al, 1990; Schott et al, 1992; King et al, 1994) have also reported similar increases in blood and normal tissue levels and tumour retention for $^{90}$Y-labelled antibodies. This resulted in all tumour to blood ratios for $^{90}$Y-labelled antibodies being slightly higher than for $^{131}$I-labelled antibodies.

However, when radiation doses estimates were corrected for equitoxic levels to the blood (Table 4), and the tumour to normal tissues ratios of radiation dose were considered, the ratios for all $^{90}$Y-labelled antibodies were lower than those for $^{131}$I-labelled antibodies. This is an interesting finding, as the biodistribution and dosimetry results had previously favoured use of $^{90}$Y-labelled antibodies based on the higher tumour to blood ratios. For RIT, efficient tumour localization is required to achieve high levels of tumour cell kill, but the corresponding dose to normal tissues is also a major consideration. Since the absorbed dose to normal tissues contributes to overall myelotoxicity, this finding suggests that the maximum tolerated dose in RIT studies will be lower for $^{90}$Y-labelled antibodies than for those labelled with $^{131}$I. The therapeutic effects of $^{131}$I-labelled antibodies were not directly compared here. But in two similar studies it was concluded that, despite the efficacious energy emissions of $^{90}$Y, IgG labelled with $^{131}$I may have equal or better therapeutic prospects than $^{90}$Y-IgG as a consequence of the higher retention of $^{90}$Y-IgG in normal organs (Buchsbaum et al, 1990; Sharkey et al, 1990).

It is unlikely that the higher radiation dose to the normal tissues after radiolabelling with $^{90}$Y is a product of conjugate instability. It is well known that free $^{90}$Y accumulates in the bone (Harrison et al, 1991), but the cumulative dose to the bone throughout this study was relatively low and not vastly different to that of other normal tissues, thus reflecting the high stability of the macrocycle conjugate. It is also unlikely that the higher radiation doses of $^{90}$Y-labelled antibodies were due to different distribution of the antibody. The same preparations of antibody fragments were used in each experiment and after radiolabelling immunoreactivity and original size characteristics were retained (Casey et al, 1996). Therefore the increase in tissue doses of $^{90}$Y-labelled antibodies is most probably due to the higher energy of and greater path length of the pure β-emitter $^{90}$Y, while $^{90}$Y is also known to be retained within cells for longer than $^{131}$I (Press et al, 1996).

The major organs contributing to the increase in absorbed dose of $^{90}$Y-labelled antibodies were the kidney, liver and spleen. The high kidney uptake level for $^{90}$Y-labelled $F(ab')_2$ and DFM was first recognized in the previous biodistribution study (Casey et al, 1996), and here the absorbed dose to the kidney for $^{90}$Y-labelled $F(ab')_2$ and DFM was increased by approximately 100-fold compared to their respective $^{131}$I-labelled fragments. Conventional radiotherapy studies have indicated that doses to this organ should not exceed 1500 cGy (Fawwaz et al, 1986), therefore cumulative kidney doses of 1065 or 1084 cGy MBq$^{-1}$ β-radiation for both $^{90}$Y-DFM and $^{90}$Y-$F(ab')_2$ respectively are clearly unacceptable for RIT.

In contrast to the divalent fragments, kidney accumulation of $^{90}$Y-TFM was greatly reduced. This is presumably mainly due to the increase in molecular weight, which theoretically restricts passage through the glomerular filter; however, there may be other factors such as shape and charge which also influence the filtration process (Sumpio and Hayslett, 1985). Nevertheless, estimated total absorbed dose to the kidney was approximately doubled on administration of $^{90}$Y-TFM (316 cGy MBq$^{-1}$) compared to $^{90}$Y-IgG (152 cGy MBq$^{-1}$), but the absorbed dose for respective $^{131}$I-labelled antibodies was fairly similar: 13.8 cGy MBq$^{-1}$ and 20.8 cGy MBq$^{-1}$. The difference is likely to be concerned with the processing of catabolic products rather than de-iodination. Radiometals such as $^{90}$Y are trapped within lysosomes whereas iodine is more readily released from cells as monoiodotyrosine (Press et al, 1996). It is also feasible that the higher stability of the thioether bond may slow down catabolism in the kidney tubules, and this effect was higher for $^{90}$Y-TFM due to the retention of this radionuclide within the cells.

 © 1999 Cancer Research Campaign

High kidney uptake of radiolabelled antibody fragments has been described in several other biodistribution studies, and this effect is more apparent with intracellularly retained radionuclides than with iodine (Sharkey et al, 1990; Behr et al, 1995; Kobayashi et al, 1996). Current research is being directed into ways of enhancing renal excretion of radiolabelled antibody fragments. One approach is based on blocking kidney tubules with cationic amino acids in order to block anionic sites and inhibit tubular reabsorption of radiolabelled fragments. A fivefold reduction in kidney uptake for $^{99m}$Tc-Fab fragments (Behr et al, 1995) and similarly a fivefold reduction for $^{177}$Lu-Fab (DePalatis et al, 1995) has been demonstrated using this approach. Other strategies include modification of the charge of antibody fragments to reduce the pI in attempt to prolong the serum half-life by retarding molecular sieving (Tarburton et al, 1990) or direct uptake by the kidney tubular cells without glomerular filtration. In a recent study by Kobayashi et al (1999) renal accumulation of Fab´ fragments increased significantly as the pI increased (pI 7–9.3). A5B7 $F(ab')_2$ and DFM both have a pI of 8, therefore reduction of the pI using techniques such as glycolation could significantly reduce the renal uptake of these fragments.

Liver and splenic uptake levels of $^{90}$Y-labelled antibodies were also elevated. In Tables 2 and 3, an 18- to 80-fold increase in liver and 10- to 95-fold increase in splenic activity compared to $^{131}$I-labelled antibodies was observed. Both the liver and spleen are organs of the reticuloendothelial system and are involved in the clearance of antibody complexes from the circulation. Therefore it was not surprising that there was a higher absorbed dose to these organs, especially for $^{90}$Y-labelled antibodies due to longer intracellular retention of the radiolabel. Both the liver and spleen are relatively radioresistant organs when compared to the kidney, being able to tolerate much higher doses of radiation before there is a possibility of permanent toxicity damage. However, the contribution to overall myelotoxicity should not be disregarded.

Considering all the combinations of antibody and radionuclide corrected for equitoxic blood levels, the highest tumour to normal tissue ratio of absorbed dose was achieved for $^{131}$I-DFM, suggesting that this would be the optimal combination for RIT. Because other ratios for $^{131}$I-labelled antibodies were only slightly lower than for $^{131}$I-DFM, it was decided to select $^{131}$I-TFM for comparative therapy experiments. The dosimetry estimates were in agreement with RIT results in that twice the activity of $^{131}$I-DFM must be administered in order to produce the same therapeutic effect as $^{131}$I-TFM. This is because the more rapid circulatory clearance of DFM compared to the higher molecular weight TFM results in lower tumour levels for the same injected activity. However, the advantage of this is that the increased tumour to blood ratio produced by DFM enables administration of larger amounts of radioantibody before a similar level of toxicity to the bone marrow is reached. Also, administration of twice the activity of DFM will result in a higher initial dose rate to the tumour, which produces the same inhibition of tumour growth as TFM, although the latter is retained in the tumour for longer. Toxicity measured by WBC in this study was minimal, implying that larger doses of both $^{131}$I-DFM and $^{131}$I-TFM may be administered to produce longer tumour regression or complete cures.

Despite the improved tumour:blood ratios for TFM, the increased estimated dose to normal tissues suggested that the most promising combination with the radionuclide $^{90}$Y appeared to be IgG. This is contrary to the conclusion reached in the previous publication that $^{90}$Y-TFM is optimal, based simply on the improved tumour to blood ratios for the biodistribution experiment (Casey et al, 1996). This confirms the importance of comparing radiation dose ratios for tumour and normal tissues as well as those for blood. After correction for equitoxic blood levels, dosimetry revealed that doses to normal tissues were in fact twice the level of IgG, producing a twofold increase in the overall tumour to normal tissue ratio. This indicates that $^{90}$Y-IgG is a more suitable alternative to $^{90}$Y-TFM. $^{90}$Y-TFM administered at twice the activity of $^{90}$Y-IgG produced slightly less WBC toxicity, but also a lower therapeutic response. This indicates that higher levels of administered activity of $^{90}$Y-TFM are required to produce a similar growth delay to $^{90}$Y-IgG, but will most likely produce higher toxicity as predicted by the dosimetry calculations. Ultimately the higher residence time of $^{90}$Y-TFM in normal tissues had a negative effect by increasing toxicity, thus reducing the total dose that may be safely administered therapeutically.

There are certain advantages of antibody fragments that should not be disregarded when selecting the most appropriate targeting entity. The Fc portion of the antibody is effectively redundant in terms of antibody binding of a carrier molecule and is also the most immunogenic part of an antibody. Thus removal of the Fc does not generally compromise immunoreactivity but does reduce overall immunogenicity. Antibody fragments are also known to be advantageous in terms of increased speed of tumour penetration, which can result in delivery of a dose that is more evenly distributed throughout the tumour mass (Yokota et al, 1992). Tumour penetration was not compared in this study, but a similar recent report by Antoniw et al (1996) demonstrated more rapid penetration of A33 TFM compared to A33 IgG.

Recent studies have focused on measurements of the microdistribution of radiolabelled antibodies within tumours. Using phosphor plate technology (Johnson et al, 1990), a three-dimensional tumour dosimetry model has been designed to measure microscopic dose measurements to study the distribution of radiolabelled antibodies and fragments within tumours over time (Flynn et al, 1998). A potential application of this and other similar models is to study the importance of binding affinity in areas of low or high antigen density within tumours. Initial experiments comparing the biodistribution of radiolabelled DFM and $F(ab')_2$ within tumour sections (xenografts) have implied that DFM was retained in viable areas of the tumour for longer than $F(ab')_2$, which was more evenly distributed throughout cellular and necrotic regions. This could be explained by the increase in functional affinity of DFM and may have implications for improved RIT.

In the previous study DFM and TFM also demonstrated faster on-rates and slower off-rates than $F(ab')_2$ and IgG (Casey et al, 1996). Since human tumours are known to have a more heterogeneous distribution of antigen compared to most human xenograft tumours, higher affinity may be advantageous in terms of improved binding to areas of low antigen density (Sung et al, 1992). Future microdosimetric analysis may reveal the true potential of improved on and off-rates for DFM and TFM in human tumours, which may further assist in selection of antibody and radionuclide for future clinical trials.

The results described here on the basis of the highest tumour to normal tissue dose ratio at equitoxic blood levels, suggests that DFM labelled with $^{131}$I is the best combination of antibody and radionuclide for future RIT. This is consistent with the conclusions in the previous publication which indicated that DFM radiolabelled with $^{131}$I would be optimal, due to a significantly faster

*© 1999 Cancer Research Campaign*

on-rate than for all other antibody forms (Casey et al, 1996). However since RIT studies showed that $^{131}$I-TFM produced a similar therapeutic response to $^{131}$I-DFM, this combination should not be disregarded. Further RIT dose escalation studies and toxicity assessment are required to select the best combination for future RIT with $^{131}$I.

Future RIT experiments should involve comparisons of $^{131}$I-DFM and $^{90}$Y-IgG, including toxicity measurements, together with microdosimetric analysis in order to select the optimal combination of antibody and radionuclide for A5B7.

### ACKNOWLEDGEMENTS

This work was funded by an MRC-link grant in association with Celltech Therapeutics Ltd, by the Cancer Research Campaign and the Ronald Raven Chair in Clinical Oncology Trust.

### REFERENCES

Antoniw P, Farnsworth APH, Turner A, Haines AMR, Mountain A, Mackintosh J, Shochat D, Humm J, Welt S, Old LJ, Yarranton GT and King DJ (1996) Radioimmunotherapy of colorectal carcinoma xenografts in nude mice with yttrium-90 A33 IgG and tri-Fab (TFM). *Br J Cancer* **74**: 513–524

Behr TM, Sharkey RM, Juweid MI, Dunn RM, Zhiliang Y, Zhang CH, Siegal JA, Gold DV and Goldenberg DM (1995) Reduction of the renal uptake of radiolabelled monoclonal antibody fragments by cationic amino acids and their derivatives. *Cancer Res* **55**: 3825–3834

Buchsbaum DJ and Order SE (1990) Bone marrow dosimetry and toxicity for radioimmunotherapy. *Antibody Immunoconj Radiopharm* **3**: 213–233

Boxer GM, Chester KA, Robson L, Keep PA, Casey JL and Begent RHJ (1995) Non-cross reactive scFv anti-CEA antibody from a combinatorial bacteriophage library. *Br J Cancer* Suppl: P139 (Abstract 59)

Casey JL, King DJ, Chaplin LC, Haines AMR, Pedley RB, Mountain A, Yarranton GT and Begent RHJ (1996) Preparation, characterisation and tumour targeting of cross-linked divalent and trivalent anti-tumour Fab´ fragments. *Br J Cancer* **74**: 1397–1405

DePalatis LR, Frazier KA, Cheng RC and Kotite NJ (1995) Lysine reduces renal accumulation of radioactivity associated with injection of the ($^{177}$Lu) a-[2-(4-aminophenyl) ethyl]-1,4,7,10-tetraaza-cyclodecane-1,4,7,10-tetraacetic acid-CC49 radioimmunoconjugate. *Cancer Res* **55**: 5288–5295

Durbin PW, Jeung N, Kullgren B and Clemons GK (1992) Gross composition and plasma and extracellular water volumes of tissues of a reference mouse. *Health Phys* **63**: 427–442

Fawwaz RA, Wang TST, Srivastava SC and Hardy MA (1986) The use of radionuclides for tumour therapy. *Nucl Med Biol* **13**: 429–436

Flynn AA, Green AJ, Boxer GM, Pedley RB and Begent RHJ (1998) A dosimetry model for the accurate characterisation of absorbed dose in mouse organs. *Br J Cancer* **78**: 39

Fowler JF (1990) Radiobiological aspects of low dose rates in radioimmunotherapy. *Int J Radiation Oncology Biol Phys* **18**: 1261–1269

Ghetie V, Poror S, Borjak J, Radu C and Matesoid D (1997) Increasing the serum persistence of an IgG fragment by random mutagenesis. *Nature Biotech* **15**: 637–640

Harrison A, Walker CA, Parker D, Jankowski KJ, Cox JPL, Craig AS, Sansom JM, Beeley NRA, Boyce RA, Chaplin L, Eaton AW, Farnsworth APH, Millar K, Millican AT, Randall AM, Rhind SK, Secher DS and Turner A (1991) The in vivo release of $^{90}$Y from cyclic and acyclic ligand antibody conjugates. *Nucleic Med Biol* **18**: 469–476

Johnson RF, Pickett SC and Barker DL (1990) Autoradiography using phosphor technology. *Electrophoresis* **11**: 355–360

King DJ, Turner A, Farnsworth APH, Adair JR, Owens RJ, Pedley RB, Baldock D, Proudfoot KA, Lawson ADG, Beeley NRA, Millar K, Millican A, Boyce BA, Antoniw P, Mountain A, Begent RHJ, Shochat D and Yarranton GT (1994) Improved tumour targeting with chemically cross-linked recombinant antibody fragments. *Cancer Res* **54**: 6176–6185

King DJ, Antonwi P, Owens RJ, Adair JR, Haines AMR, Farnsworth APH, Finney H, Lawson ADG, Lyons A, Baker TS, Baldock D, Mackintosh J, Gofton C, Yarranton GT, McWilliams W, Shochat D, Leichner PK, Welt S, Old LJ and Mountain A (1995) Preparation and preclinical evaluation of humanised A33 immunoconjugates for radioimmunotherapy. *Br J Cancer* **72**: 1364–1372

Kobayashi H, Yoo TM, Kim IS, Kim MK, Le N, Webber KO, Pastan I, Paik CH, Eckelman WC and Carrasquillo JA (1996) L-Lysine effectively blocks renal uptake of $^{125}$I or $^{99m}$Tc-labelled anti-Tac disulphide stabilised Fv fragment. *Cancer Res* **56**: 3788–3795

Kobayashi H, Nhat L, Kim IS, Kim MK, Pie JE, Palik DS, Waldmann TA, Paik CH and Carrasquillo JA (1999) The pharmacokinetic characteristics of glycolated humanized anti-Tac Fabs are determined by their isoelectric points. *Cancer Res* **59**: 422–430

Lane DM, Eagle KF, Begent RHJ, Hope-Stone LD, Green AJ, Casey JL, Keep PA, Kelly AMB, Ledermann JA, Glaser MG and Hilson AJW (1994) Radioimmunotherapy of metastatic colorectal tumours with iodine-$^{131}$-labelled antibody to carcinoembryonic antigen: phase I/II study with comparative biodistribution of intact and F(ab´)$_2$ antibodies. *Br J Cancer* **70**: 521–525

Pedley RB, Boden JA, Boden RW, Green A, Boxer GM and Bagshawe KD (1989) The effect of serum CEA on the distribution and clearance of anti-CEA antibody in a pancreatic tumour xenograft model. *Br J Cancer* **60**: 549–554

Pedley RB, Boden JA, Boden R, Dale R and Begent RHJ (1993) Comparative radioimmunotherapy using intact or F(ab´)$_2$ fragments of $^{131}$I anti-CEA antibody in a colonic xenograft model. *Br J Cancer* **68**: 69–73

Pedley RB, Boden JA, Boden R, Boxer GM, Flynn AA, Keep PA and Begent RHJ (1996) Ablation of colorectal xenografts with combined radioimmunotherapy and tumour blood flow-modifying agents. *Cancer Res* **56**: 3293–3300

Press OW, Shan D, Howell-Clarke J, Eary J, Applebaum FR, Matthews D, King DJ, Haines AMR, Hamann P, Hinmann L, Shochat D and Bernstein ID (1996) Comparative metabolism and retention of Iodine-125, Yttrium-90, and Indium-111 radioimmunoconjugates by cancer cells. *Cancer Res* **56**: 2123–2129

Schott ME, Milenic DE, Yokota T, Whitlow M, Wood JF, Fordyce WA, Cheng RC and Schlom J (1992) Differential metabolic patterns of iodinated versus radiometal chelated anticarcinoma single chain Fv molecules. *Cancer Res* **52**: 6413–6417

Sharkey RM, Motta-Hennessy C, Pawlyk D, Siegal JA and Goldenberg DM (1990) Biodistribution and radiation dose estimates for Yttrium and Iodine-labelled monoclonal antibody IgG and fragments in nude mice bearing human colonic tumour xenografts. *Cancer Res* **50**: 2330–2336

Siegel JA, Wessels BW, Watson EE, Stabin MG, Vriesendorp HM, Bradley EW, Badger CC, Brill AB, Kwok CS, Stickly DR, Eckerman KF, Fisher DR, Buchsbaum DJ and Order SE (1990) Bone marrow dosimetry and toxicity for radioimmunotherapy. *Antibody, Immunoconj Radiopharm* **3**: 213–233

Sumpio BE and Hayslett JP (1985) Renal handling of proteins in normal and disease states. *Quart J Med* **57**: 611–635

Sung C, Shockley TR, Morrison PF, Dvorak HF, Yarmush ML and Dedrick RL (1992) Predicted and observed effects of antibody affinity and antigen density on monoclonal antibody uptake in solid tumours. *Cancer Res* **52**: 377–384

Tarburton JP, Halpern SE, Hagan PL, Sudora E, Chen A, Fridman DM and Pfaff AE (1990) Effect of acetylation on monoclonal antibody ZCE-025 Fab´: Distribution in normal and tumour bearing mice. *J Biol Resp Mod* **9**: 221–230

Yokota T, Milenic DE, Whitlow M and Schlom J (1992) Rapid tumour penetration of a single chain Fv and comparison with other immunoglobulin forms. *Cancer Res* **52**: 3402–3408

 © 1999 Cancer Research Campaign
